# Rapamycin Attenuated Cardiac Hypertrophy Induced by Isoproterenol and Maintained Energy Homeostasis via Inhibiting NF-*κ*B Activation

**DOI:** 10.1155/2014/868753

**Published:** 2014-06-19

**Authors:** Xi Chen, Siyu Zeng, Jian Zou, Yanfang Chen, Zhongbao Yue, Ying Gao, Luankun Zhang, Weiwei Cao, Peiqing Liu

**Affiliations:** ^1^Department of Pharmacology and Toxicology, School of Pharmaceutical Sciences, Sun Yat-sen University, Guangzhou 510006, China; ^2^Department of Pharmacy, Chengdu Fifth People's Hospital, Chengdu 611130, China; ^3^The Second Affiliated Hospital of Guangzhou Medical University, Guangzhou 510260, China

## Abstract

Rapamycin, also known as sirolimus, is an immunosuppressant drug used to prevent rejection organ (especially kidney) transplantation. However, little is known about the role of Rapa in cardiac hypertrophy induced by isoproterenol and its underlying mechanism. In this study, Rapa was administrated intraperitoneally for one week after the rat model of cardiac hypertrophy induced by isoproterenol established. Rapa was demonstrated to attenuate isoproterenol-induced cardiac hypertrophy, maintain the structure integrity and functional performance of mitochondria, and upregulate genes related to fatty acid metabolism in hypertrophied hearts. To further study the implication of NF-*κ*B in the protective role of Rapa, cardiomyocytes were pretreated with TNF-*α* or transfected with siRNA against NF-*κ*B/p65 subunit. It was revealed that the upregulation of extracellular circulating proinflammatory cytokines induced by isoproterenol was able to be reversed by Rapa, which was dependent on NF-*κ*B pathway. Furthermore, the regression of cardiac hypertrophy and maintaining energy homeostasis by Rapa in cardiomyocytes may be attributed to the inactivation of NF-*κ*B. Our results shed new light on mechanisms underlying the protective role of Rapa against cardiac hypertrophy induced by isoproterenol, suggesting that blocking proinflammatory response by Rapa might contribute to the maintenance of energy homeostasis during the progression of cardiac hypertrophy.

## 1. Introduction

Cardiac hypertrophy was induced by many kinds of physiological and pathophysiological stimuli, including exercise, pressure or volume overload, endocrine disorders, and valvular heart diseases [1]. Although it has been viewed as a temporary compensatory mechanism for hearts to diminish wall stress, cardiac hypertrophy is associated with increased risks of developing decompensatory heart failure [2].

Hearts consume more energy than any other organs to maintain excitation-contraction coupling. As a result, common heart diseases, either myocardial ischemia or myocardial infarction, were characterized by energy metabolism dysregulation. It has been demonstrated that fatty acid utilization was decreased significantly, resulting in energy insufficiency that exacerbated the progression of heart diseases [3]. The reduction in fatty oxidation led to the abnormal accumulation of intracellular lipid, which could be lipotoxic to hearts [4]. Mitochondria provide ATP to hearts for preserving contractile and relaxation functions through conversion of metabolism substrates to usable energy [5]. Thus, it is of great importance for hearts to maintain structure integrity and functional performance of mitochondria, as well as the efficiency in utilization of fatty acid [6].

Inducements of cardiac hypertrophy are of great complexities. The retention of salt and water, as well as excessive peripheral vasoconstriction, was to blame for the disease progression. And prior to overtly symptomatic decompensatory cardiac hypertrophy, sympathetic nervous system (SNS) activation was induced to increase the cardiac output at the onset of heart disease [7]; however, the persistent stimulation of neurohormonal factors contributes to the mechanical dysfunction of hearts ultimately [8]. It had been demonstrated that overexpression of biologically active neurohormonal molecules contributed to the progression of decompensated cardiac hypertrophy independently of the hemodynamic status, by virtue of the direct toxic effects of these neurohormonal molecules exert on hearts [9]. Isoproterenol is one of these biologically active neurohormonal molecules. In addition to its direct cardiac effect, isoproterenol was proved to have a cardiac trophic effect via stimulation of the renin angiotensin system (RAS) [10].

Rapamycin is a clinically used immunosuppressor in preventing transplant rejection [11] and restenosis in coronary arteries following balloon angioplasty. Recently, Rapa was demonstrated to ameliorate cardiac remodeling and improve the left ventricular function after myocardial infarction [12]. Besides, it has been reported that Rapa regressed left ventricular hypertrophy in renal transplant recipients regardless of blood pressure changes, suggesting nonhemodynamic-effect mechanisms of rapamycin on left ventricular mass [13]. Thus, it is worthy of investigation that whether the treatment of Rapa was capable of regressing cardiac hypertrophy induced by persistent stimulation of sympathetic nervous system. And the underlying mechanisms of Rapa in protecting against cardiac hypertrophy induced by isoproterenol remain to be elucidated.

Based on these considerations mentioned above, we established the rat model of cardiac hypertrophy induced by isoproterenol, and then Rapa was administrated to evaluate the cardiac function and mitochondrial functional performance. In this study, Rapa was observed to attenuate isoproterenol-induced cardiac hypertrophy and maintain energy homeostasis in adult rats. Increased levels of proinflammatory cytokines were detected in cardiomyocytes in response to isoproterenol, which could be substantially attenuated by Rapa. Moreover, these protective effects of Rapa might be attributed to the inactivation of NF-*κ*B. The present data revealed that the role of Rapa in protecting hearts from isoproterenol-induced cardiac hypertrophy and maintaining energy homeostasis was dependent on NF-*κ*B pathway.

## 2. Materials and Methods

### 2.1. Animal Models of Cardiac Hypertrophy and Experimental Protocols

Male Sprague-Dawley rats weighing 225 g to 250 g were purchased from Animal Breeding Center of Sun Yat-sen University. Isoproterenol (5.5 mg/Kg, Merck) dissolved in saline was injected subcutaneously for consecutive ten days. Control animals underwent the same procedure, except that saline vehicle was injected subcutaneously instead of isoproterenol. After the infusion of isoproterenol, rats surviving were assigned to the following groups randomly: a vehicle-treated group (vehicle, *n* = 6), a rapamycin-treated group (1.2 mg/kg, Selleck; *n* = 6). Treatments of Rapa were injected intraperitoneally daily for seven days. The doses we administrated here were comparable with those used in mice studies when normalized by body surface area [12, 14]. The experiments were approved by the ethics Committees of Sun Yat-sen University of medical Science.

### 2.2. Echocardiographic Studies and Histological Analysis

Rats were anesthetized with 2% isoflurane allowing spontaneous breathing. The Vevo 2100 scanner equipped with a 16 MHz Probe (Visual Sonics, Toronto, Canada) was used to perform echocardiography analysis. After a good-quality two-dimensional image was obtained, M-mode images of left ventricles were recorded. Left ventricular anterior and posterior wall thickness (LVAW and LVPW), fractional shortening (LVFS), and ejection fraction (LVEF) were measured and analyzed.

Once all rats were sacrificed, the hearts were washed with PBS and dried to weigh and fixed within 4% paraform overnight. Paraffin-embedded sections (7 *μ*m) were stained with hematoxylin and eosin. Slides were examined with brightfield microscopy and ×400 images were captured. The mean myocyte diameter was calculated by randomly measuring 100 cardiomyocytes in 10 randomly chosen fields according to method and criteria reported [15, 16].

### 2.3. Transmission Electron Microscopy Analysis

Cardiac tissues were cut into 1 mm cubes immediately after sacrifice and fixed in 2.5% glutaraldehyde and then postfixed in 1% osmium tetroxide. After acetone dehydration and embedding, the ultrathin sections were stained as methods described [17]. Images were taken at 120 KV with JEM1400 electron microscopy equipped with a high-performance CCD camera.

### 2.4. Mitochondrial Oxygen Consumption

Hearts were freshly excised, and the apex of left ventricle (200 mg) was obtained from hearts in three experimental groups. Then fresh mitochondria were isolated using mitochondria extraction and isolation kit (GenMed Scientifics) according to the manufacturer's instruction. Clark-type oxygen electrode (Strathkelvin, Scotland) was used to determine the rate of mitochondrial respiration (25°C). The respiration medium was prepared as method reported [18]. Following the addition of 1 mM adenosine diphosphate (ADP), the state III respiration was recorded. After the complete phosphorylation of the added ADP, the state IV respiration was recorded as well. The respiration control rate (RCR), which reflects the function of mitochondria, was represented by the ratio of rate of state III respiration to the rate of state IV respiration. Mitochondrial protein concentration was determined by BCA protein assay kit (Thermo Scientifics). The rates of oxygen consumption were expressed as nmol O_2_/min/mg.

### 2.5. Quantitative Real-Time Polymerase Chain Reaction (qRT-PCR)

According to the manufacturer's instruction, myocardial RNA was extracted from snap-frozen tissues with Trizol reagent (Invitrogen). The SYBR-Green Quantitative PCR kit (Takara) was used to determine the mRNA expression levels of each target gene by iCycler iQ system (Bio-Rad). All PCR reactions were performed in triplicate. Specific primer sequences synthesized by Invitrogen were listed in Table 1. GAPDH and *β*-actin served as an endogenous control.

### 2.6. ATP Concentration Measurements

Using an ATP bioluminescent assay kit (Sigma), myocardial ATP concentration was measured according to the manufacturer's recommendations. Experiments were done in triplicate for each group.

### 2.7. Western Blot Analysis

Using a commercial available Nuclear and Cytoplasm Extraction Kit (Active Motif), nuclear proteins were isolated from left ventricles of rats or primary neonatal rat ventricular cardiomyocytes. The homogenate proteins were separated on SDS-PAGE and then transferred to PVDF membrane (Millipore). The membranes were incubated with primary polyclonal antibodies against p65 (Cell Signal Technology), I*κ*B-*α* (Cell Signal Technology), Histone-H3 (Sigma), p-mTOR (Cell Signal Technology), mTOR (Cell Signal Technology), PPAR*α* (Sigma), PGC-1*α* (Calbiochem), GAPDH (Sigma), and *α*-tubulin (Sigma) overnight at 4°C. After washing in TBS-T buffer, the second antibodies (Promega) were conjugated at room temperature, and then protein bands were visualized using enhanced Super-Signal West Pico substrates (Pierce).

### 2.8. Primary Culture of Neonatal Rat Ventricular Cardiomyocytes and Treatments

Primary culture of neonatal rat ventricular cardiomyocytes was prepared using 1- to 3-day-old Sprague-Dawley rats as methods we reported [19]. Cells were cultured in 10% serum for 24 hours after isolation. 48 hours later, the serum-containing culture medium was replaced with DMEM containing 0.1% FBS. After incubation for 24 hours, the cells were further treated with isoproterenol (1 *μ*mol/L), or in combination with rapamycin (100 nmol/L) was administrated to cells and maintained for 24 hours.

### 2.9. Small Interference RNA

The small interference RNA (siRNA) sequences against rat NF-*κ*B p65 subunit were synthesized by GenePharma. NF-*κ*B p65 antisense sequences were 5′-UCUAUGGGAACUUGAAAGGTT-3′; the scrambled sequence was used as a negative control (NC). Small interference RNA (100 nmol/L) was transiently transfected into cardiomyocytes by adding it to lipofectamine 2000 (Invitrogen) according to the manufacturer's instructions. The knockdown of p65 protein expression was confirmed by western blot analysis.

### 2.10. TNF-*α*, IL-1*β*, and IL-2 Measurement

An ELISA kit (Dakewe) was used to determine the concentrations of IL-1*β*, IL-2, and TNF-*α* in the culture medium according to the manufacturer's instructions.

### 2.11. Cell Surface Area Quantification

To determine the cell surface area, cells were fixed with 4% paraform and then permeabilized within 0.5% TritonX-100, after which cells were incubated with TRITC-labeled phalloidin (Sigma) for 30 min at room temperature as methods reported [20].

### 2.12. Mitochondrial Membrane Potential Analysis

TMRE (10 nmol/L), a mitochondrial potential-indicating fluorophore (Invitrogen, Molecular Probes), was added to cells for 30 min, after which cells were visualized under confocal laser scanning microscopy (LSM710, Zeiss). Images were analyzed as reported previously [21].

### 2.13. Statistical Analysis

Data were expressed as mean ± SD. All results were obtained from three independent experiments. SPSS 17.0 for statistic software was used to perform all data analyses. Statistical significance of difference among experimental groups was assessed with one-way analysis of variance (ANOVA) followed by the Bonferroni post hoc test. The level of *P* < 0.05 was considered to be statistically significant in all cases.

## 3. Results

### 3.1. Rapa Attenuated Cardiac Hypertrophy Induced by Isoproterenol in Adult Rats

**Figure 1:**
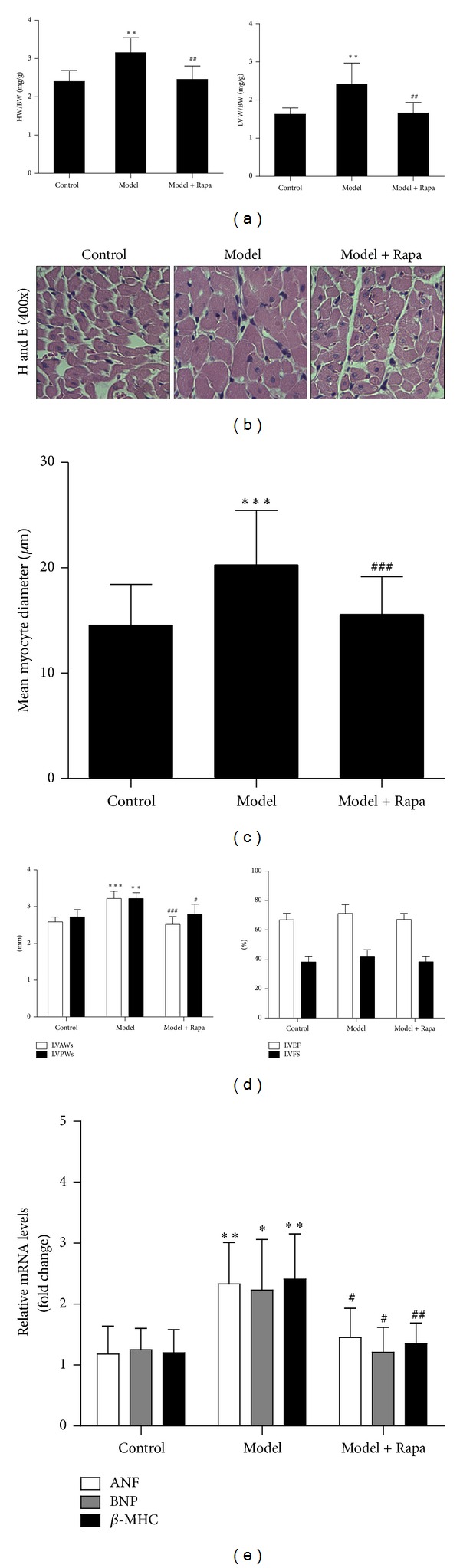
Rapa attenuated cardiac hypertrophy induced by consecutive infusion of isoproterenol. (a) Changes in the ratio of heart weight to body weight (HW/BW) and the ratio of left ventricular weight to body weight (LVW/BW). Values represented mean ± SD of 6 rats in each group. (b) Representative high magnification (×400) of left ventricular stained with hematoxylin and eosin from each group. (c) Mean myocyte diameter was calculated by randomly measuring 100 cells from 5 sections in each group. (d) The thickness of left ventricular anterior and posterior wall (left) and left ventricular fraction shortening and left ventricular ejection fraction. Echocardiographic measurements were performed, and values represented as mean ± SD of 6 rats in each group. (e) Gene expression patterns of ANF, BNP, and *β*-MHC among different groups. The mRNA was prepared and normalized to GAPDH gene. Model, the cardiac hypertrophy model induced by isoproterenol; Rapa, isoproterenol-infused rats treated with Rapa. Values represented as mean ± SD of 4 to 5 rats in each group. *P < 0.05 versus control group; **P < 0.01 versus control group; ***P < 0.001 versus control group; ^#^ *P* < 0.05 versus model group; ^##^ *P* < 0.01 versus model group; and ^###^ *P* < 0.001 versus model group.

We first determined whether the treatment of Rapa after consecutive isoproterenol-infusion for ten days reverses indices of myocardial hypertrophy. The ratios of heart weight to body weight (HW/BW) and left ventricular weight to body weight (LVW/BW) were significantly upregulated in isoproterenol-infusion model group compared with those in control group (*P* < 0.01 versus control). As shown in Figure 1(a), rats treated with Rapa reduced the isoproterenol-induced increase in the ratios of both HW/BW and LVW/BW significantly (HW/BW: 2.46 ± 0.35 versus 3.15 ± 0.39; *P* < 0.01 versus model; LVW/BW: 1.66 ± 0.28 versus 2.40 ± 0.55; *P* < 0.01 versus model). Histological analysis revealed that hearts under isoproterenol stimulation demonstrated direct evidence of increased mean myocyte diameters, which was reversed in the Rapa-treated hearts (Figures 1(b) and 1(c)). Consistently, echocardiographic analysis showed that Rapa reduced the isoproterenol-induced increases in the thicknesses of left ventricular anterior and posterior walls during systole (LVAWs, LVPWs) (LVAWs: 2.52 ± 0.21 versus 3.22 ± 0.20; *P* < 0.001 versus model; LVPWs: 2.80 ± 0.27 versus 3.22 ± 0.16; *P* < 0.05 versus model). On the other hand, the cardiac function, which was represented by LVEF and LVFS, was not changed (*P* = ns versus model) (Figure 1(d)).

Additionally, real-time PCR analysis was performed to quantify the expression of hypertrophic marker genes including ANF, BNP, and *β*-MHC (Figure 1(e)). As expected, levels of ANF, BNP, and *β*-MHC mRNA were increased about 2-fold in model group (*P* < 0.05 versus control). In contrast, Rapa treatment significantly decreased the expression of these marker genes, comparing with the model group (*P* < 0.05 versus model).

### 3.2. Effects of Rapa on the Energy Homeostasis in Hypertrophied Hearts

**Figure 2:**
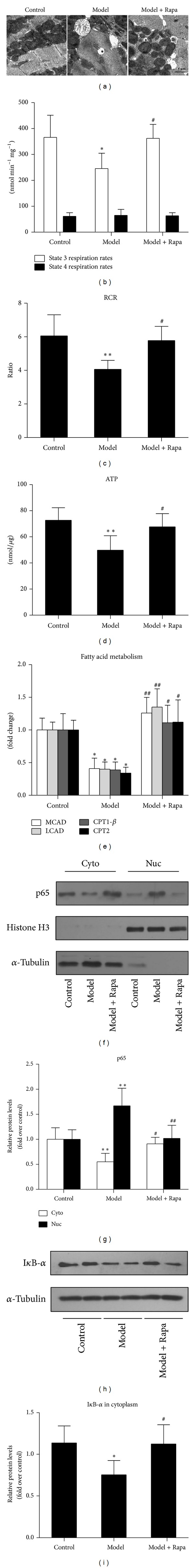
Rapa maintained energy homeostasis and inhibited NF-*κ*B activation in hypertrophied hearts induced by isoproterenol. (a) The ultrastructures of the mitochondria were analyzed under TEM. Transmission electron micrographs of histological sections from the apex of left ventricle with different treatments were shown. Black arrows indicate disorganized cristae and vacuoles of mitochondria in the hypertrophied hearts. White arrows indicate autophagosomes. Scale bars, 1 *μ*m. (b) State III and state IV respiration rates. (c) Respiratory control rate (RCR) was calculated as the ratio of state III respiration rates to state IV respiration rates. Values represented as mean ± SD of 6 rats in each group. (d) ATP production. Values represented as mean ± SD of 6 rats in each group. (e) Expression of metabolic genes associated with fatty acid metabolism at mRNA levels. The mRNA was prepared and normalized to *β*-actin gene. Values represented as mean ± SD of 4 to 5 rats in each group. ((f) and (h)) Cytoplasmic and nuclear extracts from left ventricles of three groups of rats were immunoblotted with anti-p65 and anti-I*κ*B-*α* antibodies. ((g) and (i)) Densitometric analysis of the relative protein expression of p65 and I*κ*B-*α*. Histone H3 and *α*-tubulin were used to normalize the nuclear and cytosolic proteins loading, respectively. Values represented as mean ± SD of 4 to 5 rats in each group. Model, the cardiac hypertrophy model induced by isoproterenol; Rapa, isoproterenol-infused rats treated with Rapa. *P < 0.05 versus control group; **P < 0.01 versus control group; ^#^ *P* < 0.05 versus model group; and ^##^ *P* < 0.01 versus model group.

TEM analyses were used to examine the ultrastructures of mitochondria. As seen from Figure 2(a), disorganized cristae and vacuoles of mitochondria were observed in the hypertrophied hearts; in contrast, the structure of mitochondria was well organized and the cristae stayed intact and evident in Rapa-treated hearts.

In the hypertrophied hearts treated with Rapa, state III respiration rates were significantly increased compared with those in hypertrophied hearts (*P* < 0.01 versus model). This meant the dysfunction of mitochondria in the hypertrophied hearts could be attenuated by Rapa treatment (Figure 2(b)). Consistently, the production of ATP was significantly decreased in the hypertrophied hearts infused with isoproterenol, which was substantially reversed by Rapa treatment (67.74 ± 10.04 versus 49.78 ± 11.09, *P* < 0.05 versus model) (Figure 2(d)), suggesting that the efficiency of energy supply was maintained by Rapa.

In order to investigate the role of Rapa in regulating fatty acid metabolism genes in hypertrophied hearts, we subsequently performed quantitative real time-PCR analysis among experimental groups. As shown in Figure 2(e), the treatment of Rapa prevented the declines in representative metabolic target genes associated with fatty acid metabolism, including carnitine palmitoyl transferase-1*β* and -2 (CPT-1*β* and CPT-2), and medium- and long-chain acyl-CoA dehydrogenase (MCAD and LCAD) (*P* < 0.05 versus model).

### 3.3. The Activation of NF-*κ*B Pathway in Hypertrophied Hearts Was Inhibited by Rapa Treatment

Upon activation by hypertrophic stimuli, p65, the subunit of NF-*κ*B, rapidly enters the nucleus, which resulted in elevated expression of hypertrophic genes [22]. We next examined the effects of Rapa treatment on the nuclear translocation of p65 and the degradation of I*κ*B-*α* in adult rats. The persistent stimulation of isoproterenol significantly promoted the translocation of p65 from cytoplasm to the nucleus, which were prevented by Rapa treatment (*P* < 0.05 versus model) (Figures 2(f) and 2(g)). Consistently, as shown in Figures 2(h) and 2(i), the degradation of I*κ*B-*α* was detected to increase in response to isoproterenol stimulation (*P* < 0.05 versus control), and this effect can be reversed by Rapa (*P* < 0.05 versus model).

### 3.4. Effects of Rapa on Proinflammatory Cytokines Release and Gene Expression in Cardiomyocytes via NF-*κ*B Pathway

P65 was knocked down in neonatal rat cardiomyocytes using siRNA, and TNF-*α*, a cytokine known to activate NF-*κ*B signal pathway, was used to further clarify the role of NF-*κ*B in the protective effects of Rapa in cardiomyocytes. It has been shown that the protein expression of p65 in cultured cardiomyocytes was increased by TNF-*α* in a concentration-dependent manner by western blot analysis (see Supplementary Figure 1(a) in Supplementary Material available online at http://dx.doi.org/10.1155/2014/868753), and p65 depletion was proved to decrease the protein expression of p65 (Supplementary Figure 1(c)) in cardiomyocytes.

**Figure 3:**
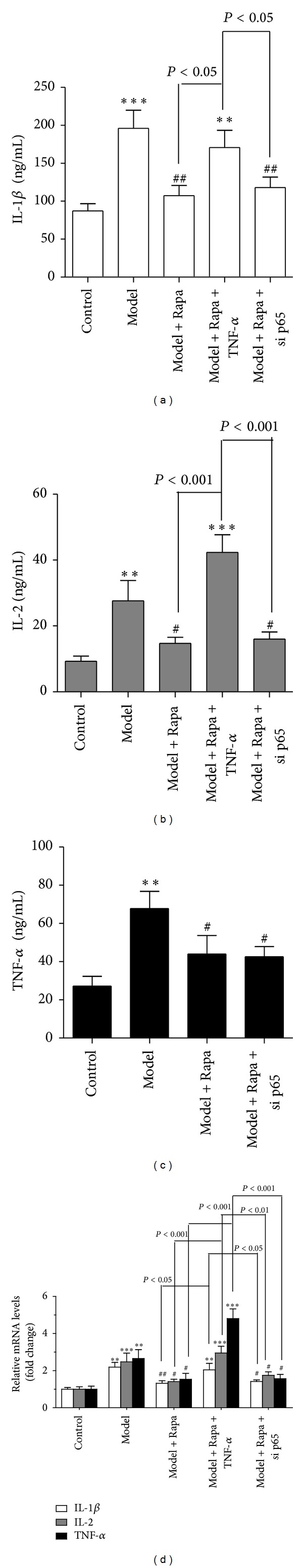
NF-*κ*B participates in the role of Rapa in reducing pro-inflammatory cytokines release and gene expressions in cardiomyocytes ((a), (b), and (c)) ELISA was used to determine the release of IL-1*β*, IL-2 and TNF-*α* into the cultured medium. (d) Real-time PCR analysis was performed to quantify the mRNA levels of proinflammatory cytokines. The mRNA was prepared and normalized to *β*-actin gene. *n* = 3 experiments in duplicate. Error bars, SD. **P < 0.01 versus control group; ***P < 0.001 versus control group; ^#^ *P* < 0.05 versus model group; and ^##^ *P* < 0.01 versus model group.

To investigate whether the activation of the NF-*κ*B pathway is implicated in proinflammatory gene expressions and cytokines releases induced by isoproterenol, the extracellular production of cytokines was measured by ELISA. As shown in Figures 3(a), 3(b), and 3(c), releases of IL-1*β*, IL-2, and TNF-*α* were significantly induced by isoproterenol (*P* < 0.01 versus control). In contrast, the administration of Rapa to cardiomyocytes attenuated the increases in releases of proinflammatory cytokines (*P* < 0.05 versus model), and this effect could be abrogated by TNF-*α* (20 ng/mL). Then real-time PCR analysis was performed to quantify the expression of representative cytokine genes. The stimulation of isoproterenol led to increases in mRNA levels of IL-1*β*, IL-2, and TNF-*α* (*P* < 0.05 versus control), which could be reversed by Rapa (Figure 3(d)). Furthermore, it was revealed that this downregulation of proinflammatory cytokines by Rapa was reversed by TNF-*α* (20 ng/mL), whereas the anti-inflammatory effect of Rapa was preserved when NF-*κ*B/p65 was depleted in cardiomyocytes.

### 3.5. NF-*κ*B Participates in the Antihypertrophic Effect of Rapa

**Figure 4:**
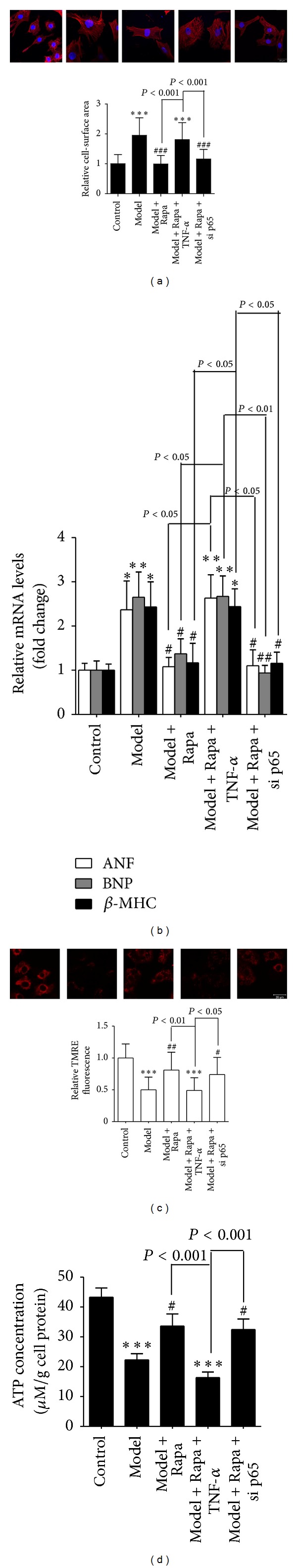
NF-*κ*B participates in the protective role of Rapa in regressing cardiac hypertrophy and energy homeostasis. (a) Cardiac myocytes were stained with rhodamine-phalloidin followed by cell surface area quantitation. (b) Gene expression pattern of atrial natriuretic factor (ANF), brain natriuretic peptide (BNP), and *β*-myosin heavy chain (*β*-MHC) among different groups. The mRNA was prepared and normalized to GAPDH gene. (c) Fluorescence of mitochondrial in TMRE loaded active cardiac myocytes under different treatments. The relative values of TMRE fluorescence intensity was normalized to 1.0. Scale bar, 20 *μ*m. The results were obtained from three independent experiments. (d) ATP production. *n* = 3 experiments in duplicate. Error bars, SD. *P < 0.05 versus control group; **P < 0.01 versus control group; ***P < 0.001 versus control group; ^#^ *P* < 0.05 versus model group; ^##^ *P* < 0.01 versus model group; and ^###^ *P* < 0.001 versus model group.

In order to explore whether the activation of the NF-*κ*B pathway is implicated in the underlying mechanism of the inhibitory effects of Rapa on cardiac hypertrophy, immunofluorescence analyses and real-time PCR analyses were performed in primary cultured neonatal cardiomyocytes. As shown in Figures 4(a) and 4(b), the surface area of cardiomyocytes, as well as mRNA levels of hypertrophied maker genes, was increased significantly in response to isoproterenol stimulation for 24 hours (*P* < 0.05 versus control). In contrast, the administration of Rapa to cardiomyocytes attenuated the increases in hypertrophy biomarkers (*P* < 0.05 versus model), and this protective effect could be abrogated by TNF-*α* (20 ng/mL). In contrast, this antihypertrophic effect of Rapa was maintained after p65 depletion in cardiomyocytes. These results suggested the involvement of NF-*κ*B inactivation in the protective effect of Rapa against cardiac hypertrophy induced by isoproterenol.

### 3.6. The Role of Rapa in Maintaining Energy Homeostasis Was Dependent on NF-*κ*B 

To investigate the role of NF-*κ*B pathway in preserving mitochondrial function in cardiomyocytes, TMRE, a sensitive fluorescent probe that reflects the level of membrane potential, were used to assess the capacity of mitochondria to produce ATP. As seen from Figure 4(c), a significant loss of membrane potential was shown in cardiomyocytes treated with isoproterenol (*P* < 0.001 versus control), which could be abolished partially by Rapa (*P* < 0.01 versus model). Consistent with TMRE staining, the ATP production in cardiomyocytes was decreased notably in response to isoproterenol stimulation, compared with vehicle control (*P* < 0.01 versus control). And this reduction in conversion of metabolic substrates into usable energy can be substantially attenuated by Rapa (*P* < 0.05 versus model) (Figure 4(d)). Moreover, it was observed that the effect of Rapa on preserving mitochondrial function and ATP production in hypertrophied cardiomyocytes was abrogated by TNF-*α* (20 ng/mL). In contrast, the protective role of Rapa in maintaining energy homeostasis was preserved after p65 depletion in cardiomyocytes.

### 3.7. The Role of Rapa in Upregulating Genes Associated with Fatty Acid Metabolism Was Dependent on NF-*κ*B

**Figure 5:**
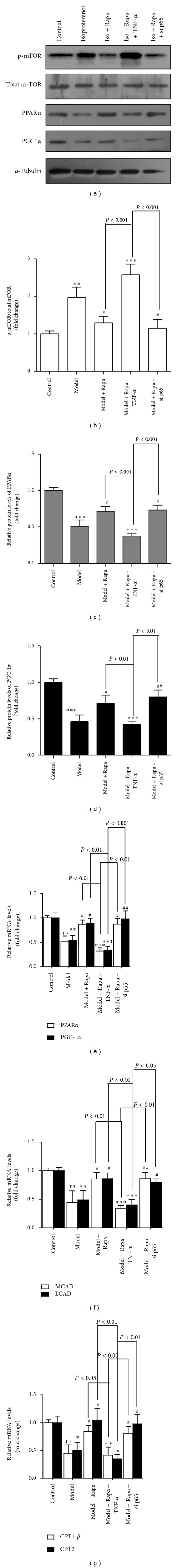
NF-*κ*B participates in the role of Rapa in upregulating fatty acid metabolism in cardiomyocytes induced by isoproterenol. (a) Cytoplasmic proteins extracted from cardiomyocytes were immunoblotted with anti-p-mTOR, mTOR, PPAR*α*, and PGC-1*α* antibodies. ((b), (c), and (d)) Densitometric analysis of the relative protein expression of the ratio of p-mTOR/mTOR, PPAR*α*, and PGC-1*α*. And *α*-tubulin was used to normalize proteins loading. ((d), (e), and (f)) Expression of metabolic genes associated with fatty acid metabolism (PPAR*α*, PGC1*α*, MCAD, LCAD, CPT-1*β*, and CPT-2) at mRNA levels. The mRNA was prepared and normalized to *β*-actin gene. *n* = 3 experiments in duplicate. Error bars, SD. *P < 0.05 versus control group; **P < 0.01 versus control group; *P < 0.001 versus control group; ^#^ *P* < 0.05 versus model group; and ^#^ *P* < 0.01 versus model group.

In order to explore the mechanisms underlying the protective role of Rapa in maintaining energy homeostasis, the activation of mTOR, which serves as a sensor of the energy state [23], was determined by western blot analysis. It was observed that the administration of isoproterenol to cardiomyocytes for 24 hours decreased the phosphorylation of mTOR at the site of serine 2448 (*P* < 0.01 versus control), which could be substantially attenuated by Rapa (Figures 5(a) and 5(b)). Furthermore, the mRNA and protein expression of its downstream transcriptional factor PPAR*α* and coactivator PGC-1*α* were determined. As shown in Figures 5(c), 5(d), and 5(e), the downregulation of PPAR*α* and PGC-1*α* in both mRNA and protein levels could be inhibited by Rapa (*P* < 0.05 versus model), and these effects of Rapa were abrogated by TNF-*α* (20 ng/mL).

Besides, the mRNA levels of medium- and long-chain acyl-CoA dehydrogenase (MCAD and LCAD) and carnitine palmitoyl transferase-1*β* and -2 (CPT-1*β* and CPT-2) were notably decreased by isoproterenol, which could be partially reversed by Rapa (*P* < 0.01 versus model); however, the effect of Rapa on upregulating these genes associated with fatty acid metabolism was abrogated by the pretreatment of TNF-*α* (20 ng/mL) (Figures 5(f) and 5(g)). Collectively, these results suggested the involvement of NF-*κ*B inactivation in the role of Rapa in upregulating genes associated with fatty acid metabolism in cardiomyocytes treated with isoproterenol.

## 4. Discussion

In this study, it was demonstrated that the regression of cardiac hypertrophy by Rapa in adult rat was associated with attenuation of the increases in myocyte cell size and HW/BW, without loss of left ventricular function. Activated NF-*κ*B pathway was detected in the hypertrophied hearts, which was substantially reversed by Rapa. Moreover, Rapa protected cardiomyocytes from the mitochondrial dysfunction and abnormal energy utilization, which could be attributed to the inactivation of NF-*κ*B. Conversely, activation of NF-*κ*B pathway resulted in the catastrophic loss of ATP production in cultured cardiomyocytes and abrogated the protective role of Rapa in regressing cardiac hypertrophy. Our results revealed the mechanisms underlying the protective role of Rapa against cardiac hypertrophy induced by isoproterenol, suggesting that blocking proinflammatory response by Rapa might contribute to the maintenance of energy homeostasis during the progression of cardiac hypertrophy.

Long-lived postmitotic cells, including neurons and cardiomyocytes, are most vulnerable to mitochondrial dysfunction [24]. In order to evaluate the effect of Rapa on the functional performance of mitochondria in hypertrophied hearts, a well-established method was performed [25]. RCR, which reflects the mitochondrial function, decreased significantly in the hypertrophied hearts induced by isoproterenol (Figure 2(c)), and these functional changes were associated with evidences of structural abnormalities in mitochondria (Figure 2(a)). And consistently, these injuries on structure and functional performance of mitochondria caused by persistent stimulation of isoproterenol could be substantially attenuated by Rapa.

NF-*κ*B had been demonstrated to be involved in pathogeneses of inflammatory disorders [26] and viewed as a link between cardiomyopathy and the dysregulation of energy metabolism [27]. In this study, we observed that the administration of Rapa to the hypertrophied hearts could reverse the activation of NF-*κ*B induced by isoproterenol (Figure 2(f)). Rapa was demonstrated to inhibit mTOR activity through a mechanism involving interaction with FKBP12 [28]. And prior to our study, this inhibition of mTOR was proved to suppress the activity of I-*κ*B kinase (an essential activator of NF-*κ*B) through a mechanism that might involve dissociation of Raptor from mTOR [29]. In keeping with these observations, we found that the degradation of I*κ*B-*α* induced by isoproterenol was substantially attenuated by Rapa administration (Figure 2(h)), thereby maintaining NF-*κ*B in an inactive state. Herein, we found that the administration of isoproterenol to cardiomyocytes led to an aberrant production of varieties of proinflammatory cytokines (Figures 3(a), 3(b), and 3(c)), which was in line with the activation of NF-*κ*B pathway induced by isoproterenol. The nuclear translocated NF-*κ*B was demonstrated to regulate the expression of various genes which encode proinflammatory cytokines including TNF-*α* [30]. Meanwhile, the downregulation of cytokine releases by Rapa was impaired under TNF-*α* treatment, indicating that the role of Rapa in restraining the inflammatory responses in cardiomyocytes under persistent stimulation of isoproterenol was NF-*κ*B-dependent.

In the present study, the stimulation of isoproterenol promoted inflammatory responses in hypertrophied cardiomyocytes and Rapa could partially restrain the downregulation of PPAR*α* induced by isoproterenol (Figure 5(a)). One potential explanation to this effect of Rapa in upregulating PPAR*α* may be its activity in inactivation of mTOR, just as we found in the decrease in the ratio of phosphor-mTOR to total mTOR (Figure 5(b)). Recently, it has been reported that pharmacological or genetic inhibition of mTORC1 was able to promote the translocation of transcription factor EB (TFEB) to the nucleus and increase its transcription activity [31]. More importantly, it was demonstrated that TFEB controls cellular lipid metabolism trough a mechanism of upregulation of PGC-1*α* and PPAR*α* [32], which were proved to play prominent roles in controlling genes mediating process from fatty acid uptake to oxidation, including medium-chain acyl-CoA dehydrogenase (MCAD) and carnitine palmitoyl transferase (CPT) [33, 34].

Herein, the present results revealed that the administration of isoproterenol to hearts weakened the expression of CPT-1*β* and CPT-2, which were proved to be crucial in mediating the rate-limiting reaction of fatty acid transport [35], and this effect could be partially abrogated by Rapa (Figure 2(e)). Moreover, clusters of genes associated with fatty acid metabolism downregulated by isoproterenol in hypertrophied cardiomyocytes were activated by Rapa, which could be abrogated by TNF-*α*. In contrast, the role of Rapa in upregulating these genes was maintained by inactivation of NF-*κ*B in cardiomyocytes (Figures 5(f) and 5(g)). Our observations reconciled with those findings concerning that TNF-*α* impaired fatty acid oxidation in the heart through activation of NF-*κ*B, which underlies cardiac dysfunction and heart failure in metabolic diseases [36].

It has been reported that the activation of S6 Kinase was critical for the development of cardiac hypertrophy in response to pressure overload. And Rapa was able to attenuate this induction of cardiac hypertrophy, which could be attributed to the inhibition of S6 Kinase [37, 38]. Similarly, previous* in vitro* studies had reported that Rapa was able to attenuate angiotensin-II-induced cardiac hypertrophy through inactivation of S6 Kinase [39]. These results indicated that the role of Rapa in regressing of cardiac hypertrophy induced by pressure-overload or angiotensin-II was associated with inactivation of S6 Kinase, which plays an important role in increases in overall protein synthesis in cardiomyocytes. In the present study, we established the model of cardiac hypertrophy induced by consecutive infusion of isoproterenol to mimic the sympathetic stimulation [40]. Herein, proinflammatory response was associated with energy metabolism alterations in the model of cardiac hypertrophy induced by isoproterenol. Rapa was demonstrated to ameliorate energy homeostasis in cardiomyocytes via inactivation of NF-*κ*B, thereby attenuating established cardiac hypertrophy after isoproterenol stimulation. These findings suggested that blocking proinflammatory response by Rapa might contribute to the maintenance of energy homeostasis and provided rational basis for development of novel strategies to prevent cardiac hypertrophy after sympathetic stimulation.

In this study, although the thickness of LVAW increased markedly in the rat model of cardiac hypertrophy induced by isoproterenol, the left ventricular function (represented by LVEF and LVFS) was maintained. One potential explanation to this paradox might be the compensatory ability of the heart, and it would develop heart failure after the infusion of isoproterenol for longer time [41]. And our findings were consistent with the reported data that there was no significant decrease in cardiac function of the hypertrophic heart [42]. Herein, we observed that during the compensated state of cardiac hypertrophy induced by isoproterenol, Rapa was able to attenuate cardiac remodeling and maintained energy homeostasis without loss of cardiac function in adult rat. This present study reconciled with the earlier findings that autophagy activated by Rapa reduced endotoxin-induced inflammatory responses in epithelial cells, maintaining intestinal homeostasis ultimately [43]. Interestingly, it has been reported that the administration of Rapa was less effective in regressing decompensated cardiac hypertrophy in mice than compensated cardiac hypertrophy (40% and 70% regression, resp.) [38]. Therefore, further studies are required to investigate mechanisms underlying the difference of Rapa in treating cardiac hypertrophy under different states.

In summary, these present* in vivo* and* in vitro* studies demonstrated the protective role of Rapa in isoproterenol-induced cardiac hypertrophy, which was dependent on NF-*κ*B pathway. These findings may provide clues to the pathophysiology of proinflammatory response associated with energy metabolism alterations during the progression of cardiac hypertrophy and shed new light on the underlying mechanism of Rapa in protecting hearts from cardiac hypertrophy under the persistent stimulation of neurohormonal factors.

## Supplementary Material

Figure S1: Effects of TNF-*α* and p65 depletion on the protein expression of p65 in cardiomyocytes (A) Cardiomyocytes were treated with TNF-*α（*0–20 ng/mL*）*and for six hours, and protein expression of p65 were detected by western blot analysis. (B) Densitometric analysis of the relative protein expression of p65 subunit in cardiomyocytes. GAPDH was used to normalize proteins loading. (C) Cardiomyocytes were incubated in transfection reagents containing 100 pmol siRNA against rat NF-*κ*B p65 subunit and scrambled sequence (Negative Control) for 48 hours according to the manufacturer's instructions, and protein expression of p65 were detected by western blot analysis. (D) Densitometric analysis of the relative protein expression of p65. GAPDH was used to normalize proteins loading. Values were presented as mean ± SD. *p < 0.05 versus Vehicle control group; **p < 0.01 versus control group.

## Figures and Tables

**Table 1:** Primer sequences for qRT-PCR.

Target gene	Sequence
ANF	Forward: 5′-GGAAGTCAACCCGTCTCA-3′
	Reverse: 5′-AGCCCTCAGTTTGCTTTT-3′
BNP	Forward: 5′-TTTGGGCAGAAGATAGACCG-3′
	Reverse: 5′-AGAAGAGCCGCAGGCAGAG-3′
*β*-MHC	Forward: 5′-GACAACGCCTATCAGTACATG-3′
	Reverse: 5′-TGGCAGCAATAACAGCAAAA-3′
GAPDH	Forward: 5′-AGGAGTAAGAAACCCTGGAC-3′
	Reverse: 5′-CTGGGATGGAATTGTGAG-3′
CPT-1*β*	Forward: 5′-TCAAGGTTTGGCTCTATGAGGGCT-3′
	Reverse: 5′-TCCAGGGACATCTTGTTCTTGCCA-3′
CPT-2	Forward: 5′-TCCTGCATACCAGCAGATGAACCA-3′
	Reverse: 5′-TATGCAATGCCAAAGCCATCAGGG-3′
MCAD	Forward: 5′-CTGCTCGCAGAAATGGCGATGAAA-3′
	Reverse: 5′-CAAAGGCCTTCGCAATAGAGGCAA-3′
LCAD	Forward: 5′-AATGGGAGAAAGCCGGAGAAGTGA-3′
	Reverse: 5′-GATGCCGCCATGTTTCTCTGCAAT-3′
IL-1*β*	Forward: 5′-TCCTCTGTGACTCGTGGGAT-3′
	Reverse: 5′-TCAGACAGCACGAGGCATTT-3′
IL-2	Forward: 5′-CCAAGCAGGCCACAGAATTG-3′
	Reverse: 5′-TCCAGCGTCTTCCAAGTGAA-3′
TNF-*α*	Forward: 5′-TGGCGTGTTCATCCGTTCTC-3′
	Reverse: 5′-CCCAGAGCCACAATTCCCTT-3′
PGC-1*α*	Forward: 5′-ACGAAAGGCTCAAGAGGGACGAAT-3′
	Reverse: 5′-CACGGCGCTCTTCAATTGCTTTCT-3′
PPAR*α*	Forward: 5′-AGCTCAGGACACAAGACGTTGTCA-3′
	Reverse: 5′-AGGGACTTTCCAGGTCATCTGCTT-3′
*β*-actin	Forward: 5′-TCGTGCGTGACATTAAAGAG-3′
	Reverse: 5′-ATTGCCGATAGTGATGACCT-3′

## Abbreviations

Rapa: Rapamycin
mTOR: Mammalian target of rapamycin
HW: Heart weight
LVW: Left ventricle weight
BW: Body weight
LVEF: Left ventricular ejection fraction
LVFS: Left ventricular fractional shortening
LVAW: Left ventricular anterior wall
LVPW: Left ventricular posterior wall
RCR: Respiratory control rate
ANF: Atrial natriuretic factor
BNP: Brain natriuretic peptide
MHC: Myosin heavy chain
NF-*κ*B: Nuclear factor-kappa B
TEM: Transmission electron microscopy
FBS: Fetal calf serum
DMEM: Dulbecco Modified Eagle Medium
TRITC: Tetraethyl rhodamine isothiocyanate
TNF-*α*: Tumor necrosis factor-*α*
TMRE: Tetramethylrhodamine ethyl ester
PPAR: Peroxisome proliferator-activated receptor
PGC-1*α*: PPAR*γ* coactivator-1*α*.
