# Supplementary figures and images for: Rapamycin Attenuated Cardiac Hypertrophy Induced by Isoproterenol and Maintained Energy Homeostasis via Inhibiting NF-κB Activation
Source: Mediators Inflamm. 2014 Jun 19;2014:868753. doi: 10.1155/2014/868753 (PMC4089551; doi:10.1155/2014/868753)

Fig. S1

A

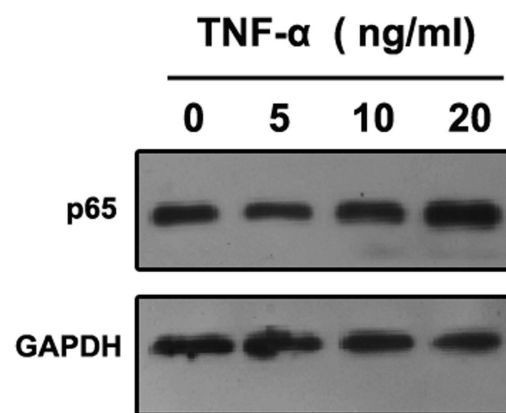

B

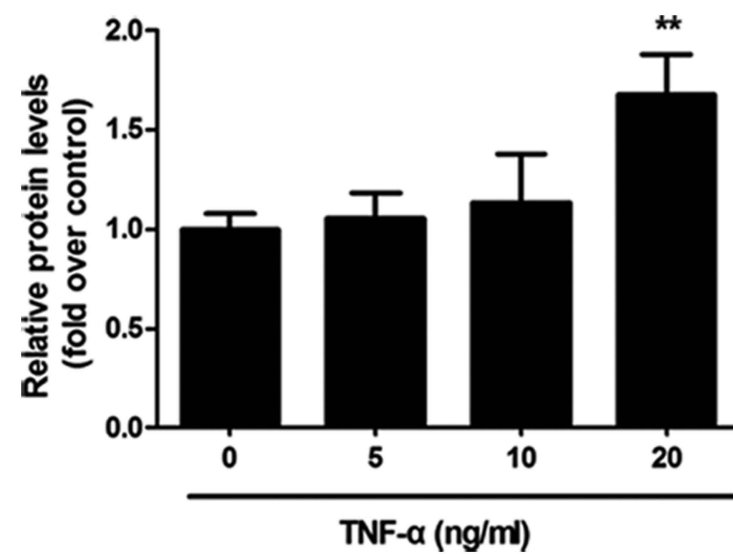

C

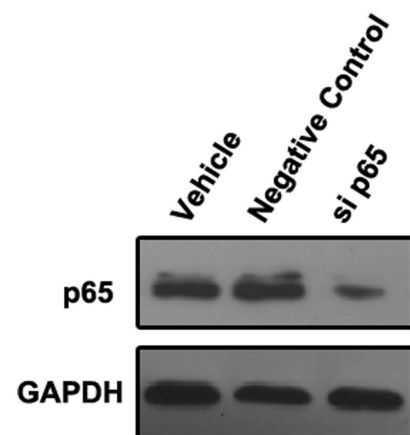

D

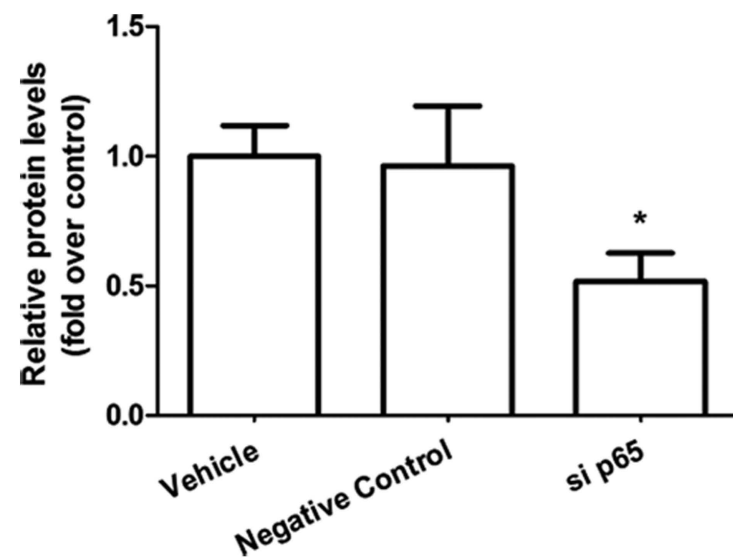

Supplement: Supplementary file 1 — Figure S1: Effects of TNF-α and p65 depletion on the protein expression of p65 in cardiomyocytes (A) Cardiomyocytes were treated with TNF-α（0–20 ng/mL）and for six hours, and protein expression of p65 were detected by western blot analysis. (B) Densitometric analysis of the relative protein expression of p65 subunit in cardiomyocytes. GAPDH was used to normalize proteins loading. (C) Cardiomyocytes were incubated in transfection reagents containing 100 pmol siRNA against rat NF-κB p65 subunit and scrambled sequence (Negative Control) for 48 hours according to the manufacturer's instructions, and protein expression of p65 were detected by western blot analysis. (D) Densitometric analysis of the relative protein expression of p65. GAPDH was used to normalize proteins loading. Values were presented as mean ± SD. *p < 0.05 versus Vehicle control group; **p < 0.01 versus control group. [file 868753.f1.pdf]
